# The concept of developmental anatomy: the greater omentum should be resected in right-sided colon cancer?

**DOI:** 10.1186/s12893-023-02020-8

**Published:** 2023-05-17

**Authors:** Kai Li, Fengyu Cao, Xiaobo He, Yongbin Zheng

**Affiliations:** Department of Gastrointestinal Surgery, Renmin Hospital of Wuhan University, Wuhan, Hubei China

**Keywords:** Developmental anatomy, Complete mesocolic excision, Right-sided colon cancer, Laparoscopic surgery

## Abstract

**Background:**

The greater omentum is derived from the foregut, and the right hemicolon is derived from the midgut based on developmental anatomy. This study aimed to investigate whether the greater omentum should be resected in laparoscopic complete mesocolic excision based on developmental anatomy for right-sided colon cancer.

**Methods:**

A total of 183 consecutive patients with right-sided colon cancer were recruited in this study between February 2020 and July 2022. Ninety-eight patients underwent standard laparoscopic complete mesocolic excision surgery (CME group). The presence of isolated tumor cells and micrometastases was detected in resected greater omentum by the HE staining and immunohistochemistry analysis. Based on developmental anatomy, laparoscopic CME surgery with greater omentum preservation (DACME group) was proposed and performed on 85 right-sided colon cancer patients. To overcome selection bias, we performed a 1:1 match between two groups using four variables: age, sex, BMI, and ASA scores.

**Results:**

No isolated tumor cells and micrometastases were found in the resected greater omentum specimen in the CME group. After the propensity score, 81 pairs were balanced and analyzed. Patients in the DACME group showed shorter operative time (194.9 ± 16.4 min vs.201.5 ± 11.5 min, *p* = 0.002), less blood loss (23.5 ± 24.7 ml vs.33.6 ± 26.3 ml, *p* = 0.013), and the shorter hospital stays (9.6 ± 1.7 days vs.10.3 ± 2.0 days, *p* = 0.010) compared with patients in the CME group. In addition, patients in the DACME group had a lower incidence of postoperative complications (4.9% vs.14.8%, *p* = 0.035) than patients in the CME group.

**Conclusion:**

The greater omentum should be preserved during right-sided colon cancer surgery, laparoscopic CME surgery based on developmental anatomy is technically safe and feasible for right-sided colon cancer.

**Supplementary Information:**

The online version contains supplementary material available at 10.1186/s12893-023-02020-8.

## Background

During embryological development, the midgut grows in length and forms the primary intestinal loop. The cephalic limb of the loop develops into the distal part of the duodenum and the small intestine, while the caudal stem develops into the right hemicolon. The dorsal mesogastrium bulges out to form the greater omentum and omental bursa, and the greater omentum expands rightward. The posterior layer of the greater omentum fuses with the anterior wall of the transverse mesocolon. The transverse mesocolon fused with the greater omentum covers the frontal surface of the dorsal meso-duodenum and meso-pancreas by the 12th week of pregnancy [1–3]. Based on embryonic development, the greater omentum is derived from the foregut, and the right hemicolon and mesocolon are derived from the midgut. Therefore, the greater omentum should be preserved theoretically in right hemicolectomy.

Currently, complete mesocolic excision (CME) has been accepted as the optimal surgical approach for right-sided colon cancer, resulting in good oncologic outcomes. It has been the standard procedure for colon cancer [4]. However, CME seems to be a theory of en-bloc resection. The greater omentum was recommended to be resected in standard CME surgery, which has been associated with certain technical challenges, mainly when performed by inexperienced surgeons. In addition, the greater omentum has long been recognized as a vibrant immunologic organ, which is essential in fighting intra-abdominal infections [5]. Besides, the greater omentum resection not only increases the operative time and intraoperative blood loss but may also lead to a higher incidence of complications such as incisional infections, abdominal abscesses, ileus, and mesocolonic injuries [6–9]. A multi-center prospective cohort study also recommended that greater omentum resection should be omitted in radical (sub)total gastrectomy for gastric cancer [10]. In the present study, we applied the concept of embryonic development to surgical anatomy to form a new concept of developmental anatomy. Subsequently, we further described the clinical application of developmental anatomy in laparoscopic complete mesocolic excision (DACME) surgery. This study aimed to investigate whether the greater omentum should be resected in laparoscopic CME for right-sided colon cancer and propose a novel laparoscopic CME surgery based on developmental anatomy.

## Materials and methods

### Patients

A total of 183 consecutive patients with right-sided colon cancer undergoing laparoscopic CME in the department of gastrointestinal surgery, Renmin Hospital of Wuhan University were recruited in this study between February 2020 and July 2022. The inclusion criteria were as follows: (1) Right-side colon cancer was confirmed pathologically by endoscopic biopsy, (2) American Society of Anesthesiologists (ASA) scores were 1–3, (3) The tumor was confirmed without serosal invasion by the final pathological report (4) Laparoscopic radical resection was performed, and (4) No pelvic or distant metastasis was found. The exclusion criteria were: (1) emergency surgery due to the complication (bleeding, obstruction, or perforation) caused by colon cancer, and (2) pregnant or breastfeeding. Patients who underwent standard laparoscopic CME surgery were divided into the CME group. Those who underwent laparoscopic CME surgery with greater omentum preservation based on developmental anatomy were divided into the DACME group. To overcome selection bias, we performed a 1:1 match between two groups using four variables: age, sex, body mass index (BMI), and ASA scores. Patient clinical characteristics and perioperative data were recorded, which included age, sex, BMI, ASA scores, tumor location, tumor differentiation, pathological TNM stage, operative time, estimated blood loss, postoperative hospital stay, lymph node harvested, postoperative complication, and major complication. According to the Clavien-Dindo classification system, minor complications were defined as grade I–II events, and major complications were defined as grade ≥ III complications [11]. This study was approved by the institutional review board of the Renmin Hospital of Wuhan University.

## Samples

**Fig. 1:**
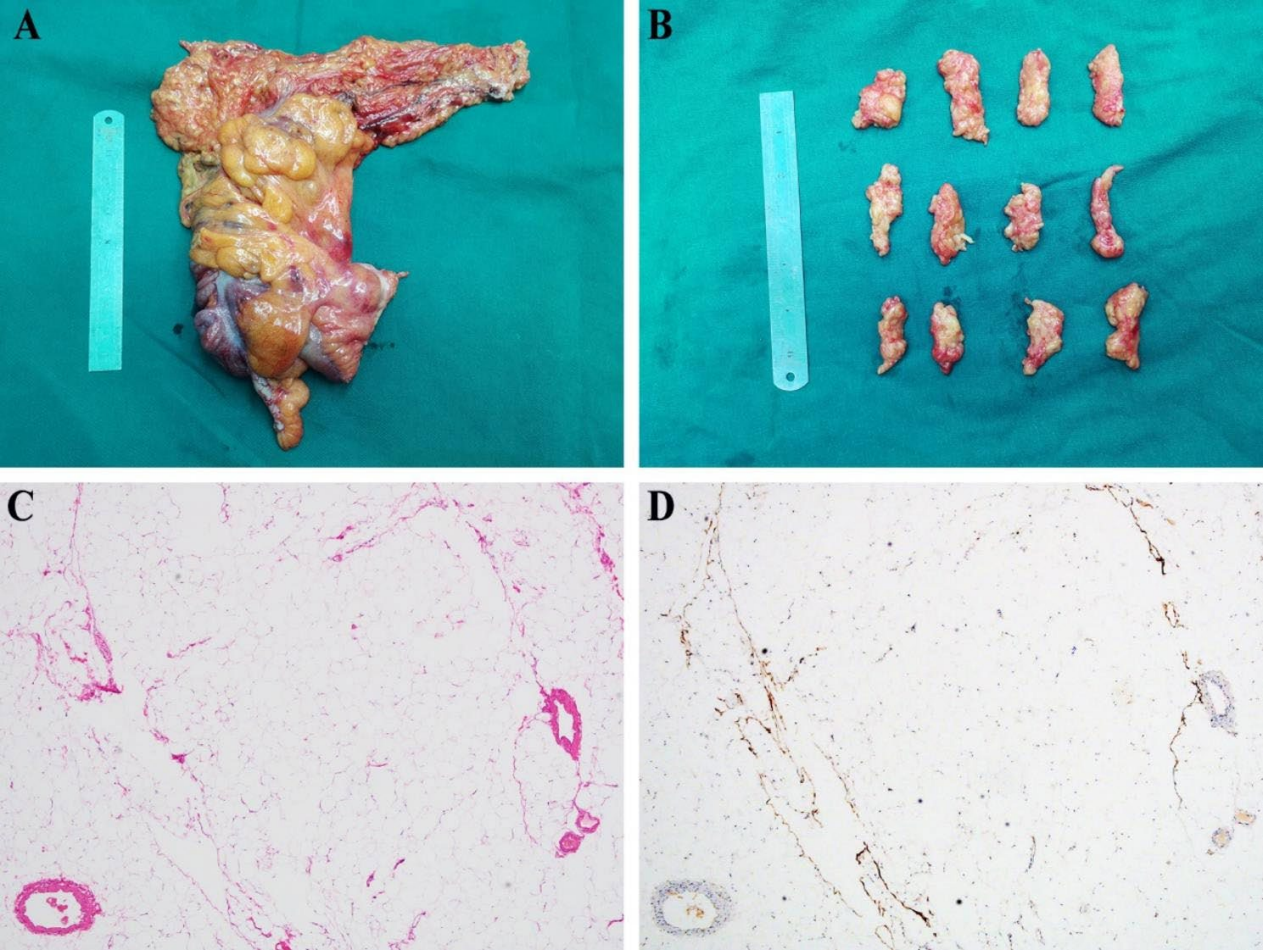
Detection of micrometastasis and isolated tumor cells in the greater omentum specimen **A**: The resected right hemicolon specimen **B**: Continuous sections at 1.5-cm-width intervals of the greater omentum specimen **C**: HE staining of the greater omentum specimen **D**: Immunohistochemistry analysis of the greater omentum specimen

Patients in the CME group underwent standard laparoscopic CME with the greater omentum resection, and the resected greater omentum specimens were cut into cubes at 1.5-cm-width intervals **(**Fig. 1A and B**)**. These cubes were put into embedding cassettes, stored in 10% neutral-buffered formalin, and sent to a pathologic laboratory for large serial sectioning analysis. Subsequently, these cubes were subjected to hematoxylin–eosin staining, and further immunohistochemistry analysis were examined using the monoclonal antibody for anti-cytokeratin (CK; Abcam, 1: 1000, Cambridge, MA, USA) to identify micrometastasis (MMs) and isolated tumor cells (ITCs), which has been used to identify MMs and ITCs in colorectal cancer [12–14].

## Surgical technique

**Fig. 2:**
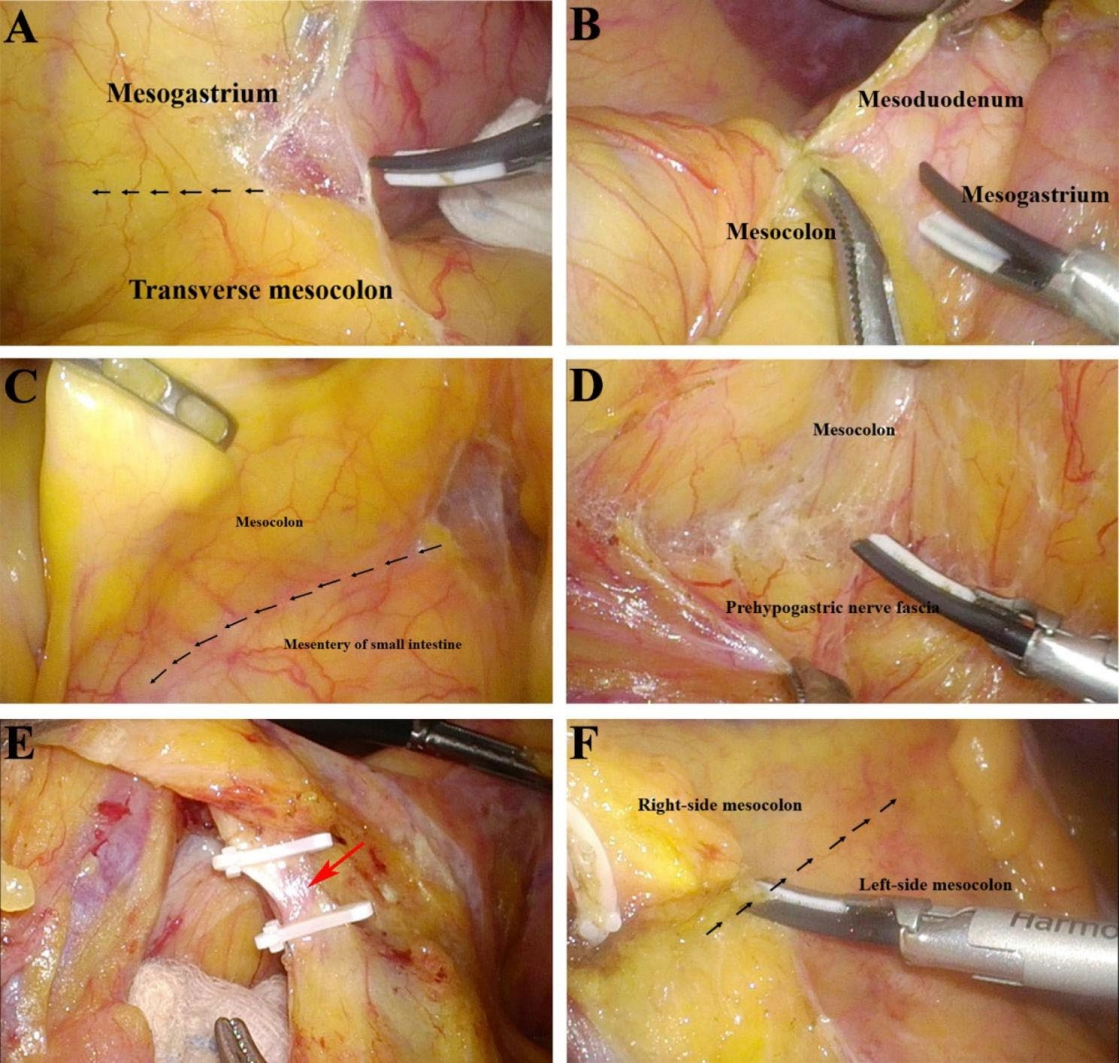
The surgical procedure laparoscopic CME surgery with greater omentum preservation based on the concept of developmental anatomy **A**: Separation of the mesogastrium and the transverse mesocolon **B**: Separation of the mesocolon from the dorsal mesoduodenum **C**: Separation of the mesocolon from the mesointestin **D**: Separation of the mesocolon from the prehypogastric nerve fascia **E**: Ligation of intra-mesenteric vessels **F**: Separation of the right-side mesocolon from the left-side mesocolon

Most surgeons were well-known standard laparoscopic CME. The details of surgical procedures of laparoscopic CME surgery with the greater omentum preservation based on the concept of developmental anatomy are as follows: Patients were first placed in the Trendelenburg position, after which five trocars were inserted (infraumbilical area: 10 mm optic, right lower quadrant: 5 mm, right upper quadrant: 5 mm, left upper quadrant: 10 mm, left lower quadrant: 5 mm). Carbon dioxide was inflated through the intraumbilical trocar, and the pressure was maintained at 15 mmHg. The operating surgeon stood on the patient’s left side, with the assistant on the right. (1) Cephalic dissection: As shown in the attached **Supplementary Video 1**, the mesogastrium, the greater omentum, the meso-duodenum, and the meso-pancreas are derived from the foregut, and the right-side colon is derived from the midgut. Therefore, the cephalad separation of the right-side colon mainly relies on the separation of the midgut from the foregut. The extra-omentum approach was preferred, and the greater omentum derived from the foregut was preserved because the serosa was not invaded. The dissection plane between the mesogastrium and continuous mesentery of the gastric-transverse colon was entered and extended. The upper mesogastrium and the inferior transverse mesocolon were left intact and smooth **(**Fig. 2A). And then, the dissection continued down to the right side of descending duodenum along the mesocolic space between the mesocolon and dorsal mesoduodenum (Fig. 2B). Therefore, the dorsal mesogastrium, transverse mesocolon, and dorsal mesoduodenum were intact. (2) Dorsal separation: As shown in the attached **Supplementary Video 2**, the neuroectoderm inserted the space of mesocolon between retroperitoneum and formed the prehypogastric nerve fascia, which was the critical surgical plane in total mesorectal excision with pelvic autonomic nerve preservation for rectal cancer. Therefore, the dorsal separation of the right-side colon mainly relies on the separation of the midgut from the neuroectoderm. After exposing the dorsal aspect of the Treitz ligament, the fusion fascia between the mesointestin and the dorsal right-side mesocolon was separated along the superior part of the Treitz ligament (Fig. 2C). And then, the dissection continued upward to the right side of descending duodenum along the mesenteric fused fascia space between the prehypogastric nerve fascia and mesocolon **(**Fig. 2D**)**, and the duodenum and its dorsal mesentery were revealed. Further cephalad separation along the dorsal mesentery of the duodenum was performed, naturally extending to the continuous dorsal mesentery of the duodenum-pancreas. Finally, the dorsal dissection met with cephalic dissection. Therefore, the lower prehypogastric nerve fascia, middle mesoduodenum, upper mesopancreas, and right-side mesocolon were intact. (3) Ventral dissection. As shown in the attached **Supplementary Video 3**, a sloping natural fold can be identified by traction to the ileocolic vessels. Dissection was performed along the mesenteric fused fascia between the small intestinal and right hemicolon. Finally, the ventral dissection met with dorsal dissection. (4) Ligation of intra-mesenteric vessels and the separation of right-side mesocolon from left-side mesocolon: As shown in the attached **Supplementary Video 4**, intra-mesenteric vessels were defined and located within the fused fascia or mesocolic space, which are ligated along the mesenteric fused fascia space regardless of their name (Fig. 2E), and the vascular sheath of the superior mesenteric vein (SMV) is not damaged. And then, the right-side mesocolon was separated from the left-side mesocolon along the mesocolic space (Fig. 2F), and en-bloc resection of the right hemicolon and its mesentery was performed.

### Statistical analysis

All data are presented as mean (± SD) for continuous variables and frequency (%) for categorical variables. The independent t-test was used for continuous variables, and the Chi-square test or Fisher’s exact test for categorical variables. The survival curves were analyzed by Kaplan–Meier analysis and plotted in R software (version 4.0.3). A *p*-value < 0.05 was considered statistically significant. All statistical analyses were performed with SPSS version 26.0 for Windows (SPSS Inc., Chicago, IL, USA).

## Results

### Patient characteristics

A total of 183 consecutive patients met the inclusion and exclusion criteria in this study, 98 patients underwent standard laparoscopic CME surgery, and 85 patients underwent laparoscopic CME surgery with greater omentum preservation based on developmental anatomy. After the propensity score, 81 pairs of patients in both two groups were balanced and analyzed. Patient clinical characteristics and perioperative data between the two groups are shown in Table 1. Patients in the DACME group showed shorter operative time (194.9 ± 16.4 min vs.201.5 ± 11.5 min, *p* = 0.002), less blood loss (23.5 ± 24.7 ml vs.33.6 ± 26.3 ml, *p* = 0.013), and the shorter hospital stays (9.6 ± 1.7 days vs.10.3 ± 2.0 days, *p* = 0.010) compared with patients in the CME group. In addition, patients in the DACME group had a lower incidence of postoperative complications (4.9% vs.14.8%, *p* = 0.035) than patients in the CME group. No major complications were found in the DACME group. At the same time, there was one case with major complications (1.2%) in the CME group, diagnosed with postoperative abdominal bleeding, recovered, and discharged from the hospital after reoperation under general anesthesia. Eleven minor postoperative complications (13.6%) occurred in the CME group, which included two incisional infection cases, two postoperative ileus cases, one anastomotic leakage case, three diarrhea cases, and two chyle leak cases. Four minor postoperative complications (4.9%) occurred in the DACME group, including one incisional infection case, one postoperative ileus case, and two postoperative pneumonia cases. Patients with minor postoperative complications also recovered and were discharged after conservative treatment in both groups, and patients in the CME group with major postoperative complications also recovered after reoperation. Other clinical variables, including age, sex, BMI, ASA scores, tumor location, lymph node harvested, tumor differentiation, pathological TNM stage, and major and minor complication in the two groups, were no significant differences.

**Table 1:** Perioperative data of participants between the DACME and CME Group

Variable	DACME group(n = 81) n (%) or Mean ± SD	CME group (n = 81) n (%) or Mean ± SD	*p*
**Age (years)**	55.2 ± 6.7	55.1 ± 6.1	0.873
**Sex**			0.875
Male	42 (51.9%)	43 (53.1%)	
Female	39 (48.1%)	38 (46.9%)	
**BMI (kg/m** ^**2**^ **)**	22.3 ± 3.3	22.5 ± 3.2	0.691
**ASA score**			0.729
I	27 (33.3%)	31 (38.3%)	
II	42 (51.9%)	37 (45.7%)	
III	12 (14.8%)	13 (16.0%)	
**Tumor location**			0.530
Caecum	18 (22.2%)	15 (18.5%)	
Ascending colon	31 (38.3%)	25 (30.9%)	
Hepatic flexure	27 (33.3%)	33 (40.7%)	
Transverse colon (right third)	5 (6.2%)	8 (9.9%)	
**Operative time (min)**	194.4 ± 16.4	201.5 ± 11.5	0.002
**Blood loss (ml)**	23.5 ± 24.7	33.6 ± 26.3	0.013
**Postoperative hospital stay (d)**	9.6 ± 1.7	10.3 ± 2.0	0.010
**Lymph node harvested**	23.6 ± 9.1	21.0 ± 9.5	0.077
**Tumor differentiation**			0.096
Well and Moderate	63 (77.8%)	71 (87.7%)	
Poor and other	18 (22.2%)	10 (12.3%)	
**Pathology TNM stage** ^**†**^			0.801
I	7 (8.6%)	8 (9.9%)	
II	43 (53.1%)	46 (56.8%)	
III	31 (38.3%)	27 (33.3%)	
**Major complication**	0 (0%)	1 (1.2%)	1.000^*^
Abdominal bleeding	0	1	
**Minor complication**	4 (4.9%)	11 (13.6%)	0.058
Incisional infections	1	2	
Postoperative ileus	1	2	
Postoperative pneumonia	2	1	
Anastomotic leakage	0	1	
Diarrhea	0	3	
Chyle leak	0	2	
**Total complication**	4 (4.9%)	12 (14.8%)	0.035

SD, standard deviation

^†^ TNM stage according to the American Joint Committee on Cancer, 7th ed

^*^Fisher’s exact test

### Histopathological results

As shown in HE staining and immunohistochemistry analysis, abundant adipocytes are present, and No MMs and ITCs were observed in the resected greater omentum specimen in the CME group. Besides, no positive lymph nodes were observed in the same slide **(**Fig. 1C and D**)**.

## Discussion

The concept of CME was first introduced as the standard surgical procedure for colon cancer by professor Hohenberger according to the concept of TME for rectal cancer in 2009 [15]. It was emphasized that the sharp dissection should be performed along the mesocolon fascial plane to remove the entire mesocolon and all potential routes of metastatic tumor spread, which could reduce the local recurrence rate. The mesocolon contains the colon’s vascular and lymphatic drainage systems; therefore, adequate clearance theoretically has the same oncologic benefit as TME surgery in rectal cancer. However, the prognosis of patients after CME in colon cancer has not been improved to the same extent as patients after TME in rectal cancer [4, 16], which may be related to the following two reasons: (1) No specific ligation site for central vessel ligation, which can easily undermine the integrity of the mesocolon. (2) No clear border of right hemicolectomy, which is difficult to form a standard operating procedure.

Developmental anatomy in embryology is the branch of anatomy that studies the structural changes in individuals from fertilization. Applying the concept of developmental anatomy to surgical anatomy is more compatible with the theory of CME, which can achieve the complete dissection of the mesocolon along the embryological plane and more accurately describe the boundaries of the “envelope-like” structures. Therefore, the concept of developmental anatomy helps form a standard operating procedure for CME surgery. In the present study, compared with patients who underwent traditional laparoscopic CME, patients undergoing laparoscopic CME surgery with greater omentum preservation based on developmental anatomy not only showed shorter operative time, blood loss, and hospital stays but also lower incidence of postoperative complications might be related to the following reasons: (1) greater omentum preservation could reduce operation time and avoid the intraoperative blood loss due to the greater omentum resection. (2) greater omentum preservation could aggregate inflammatory and stem cells and fight intra-abdominal infections. Therefore, it could reduce inflammatory reactions, decrease postoperative complications, and shorten hospital stays. (3) based on developmental anatomy, dissection was performed along the mesenteric space between the prehypogastric nerve fascia and mesocolon, and all intra-mesenteric vessels were ligated within the fused fascial space. Therefore, it was also conducive to intraoperative bleeding and postoperative complications.

Several studies also demonstrated the most appropriate extent of lymph node dissection for right-sided colon cancer surgery. Kim et al. [17] reported that there was no significant difference in 3-year disease-free survival rates between D2 and D3 lymph node dissection for clinical stage I right colon cancer patients. Japanese Society for Cancer of the Colon and Rectum guidelines [18] also believed that D3 lymph node dissection is a technically demanding procedure, it is recommended to perform D2 lymph node dissection if there is no survival benefit. However, Huang et al. [19] reported that intermediate or main lymph node metastases had a poor prognosis than pericolic lymph node metastasis for colorectal cancer patients with the T_1 − 2_ stage, and overall survival rates in colon patients with pT_3_ and pT_4_ stages were significantly higher for D3 lymph node dissection than that for D2 lymph node dissection [20]. Therefore, most studies believed that the appropriate extent of lymph node dissection should be based on accurate clinical staging, but the current clinical staging is not accurate enough to make decisions on D2 or D3 lymph node dissection for colon cancer surgery. In addition, Marek et al. [21] also believed that routine removal of further lymph node stations is not recommended for pancreatoduodenectomy because survival rates are not improved but the risk of complications is greater. In the present study, we believed that the intra-mesenteric lymph node was considered a regional lymph node and should be cleaned, and the greater omentum belongs to extra-mesenteric tissue and should be preserved. There might be a possibility of tumor metastasis might break through the mesenteric boundary for colon cancer with serosal invasion, hence patients with pT_4_ stage (serosal invasion) were excluded in the present study. Therefore, we recommended the greater omentum should be preserved based on developmental anatomy if the tumor was intraoperatively confirmed without serosal invasion.

Currently, numerous studies have proposed various approaches for laparoscopic CME surgery improvement in right-side colon cancer. Completely medial access by page-turning approach [22], retroperitoneal approach [23], caudal-to-cranial approach [24], and cranial–caudal–medial counterclockwise approach [25] have been successively introduced in laparoscopic right hemicolectomy, which could be used as an alternative approach in laparoscopic right hemicolectomy. Luo et al. [26] also developed a new medial approach of vessel management, which focused on vascular dissection in two aspects. The first aspect was to divide the sheath of the superior mesentery vein from medial to lateral and reveal the roots of the main vessels, and the second aspect was to selectively ligate the branch of the gastrocolic trunk of Henle (GTH) after expanding the left and right space of GTH. There are also several different opinions about the preferred approach in right hemicolectomy. The “SMV-First” approach [27–29] was known by most surgeons, which used SMV as the starting point for initiated dissection, and vascular dissection was also performed along the anterior SMV plane. Besides, several studies also proposed “Superior Mesenteric Artery-First” approaches [30, 31] in laparoscopic right hemicolectomy, which recommends that the Treitz’s ligament and ileocolic vascular pedicle be used as the anatomical landmarks for SMA identification and exposure. These different approaches were designed to improve laparoscopic right hemicolectomy further and proved technically feasible and safe for right colon cancer. The above methods focused on vascular management, including superior mesenteric artery, SMV, and GTH dissection. In the present study, all intra-mesenteric vessels would not be actively exposed and were ligated within the fused fascial space as long as the whole dissection was based on developmental anatomy. Therefore, laparoscopic CME surgery with greater omentum preservation based on the concept of developmental anatomy could also be applied to these above different approaches.

The present study proposed laparoscopic CME surgery with greater omentum preservation based on developmental anatomy for right-sided colon cancer. The greater omentum hangs down from, the greater curvature of the stomach in front of the transverse colon and fuses with the transverse mesocolon. The greater omentum is derived from the foregut, which is unnecessary to resect in the right hemicolectomy for patients without serosal invasion. In addition, the surgical technique based on the concept of developmental anatomy also has several other differences compared with standard CME surgery, which are as follows: (1) Not only the right-side mesocolon is intact, but also the mesogastrium, the transverse mesocolon, the prehypogastric nerve fascia, the mesoduodenum, the mesopancreas, mesointestin, and left-side mesocolon was fully preserved after en-block resection; (2) This surgical technique has a clear border of right hemicolectomy (Cephalic side: the transverse mesocolon. Dorsal side: the prehypogastric nerve fascia, the mesoduodenum, and the mesopancreas. Medial side: left mesocolon. Lateral side: parietal pelvic fascia); (3) CME requires a central vascular ligation (CVL) of the trunk vessels, which might lead to bleeding and loss of surgical anatomy during the CVL procedure [32, 33]. The excessive dissection of the CVL may damage the integrity of the resected mesocolon. In addition, root ligation of CVL and vascular sheath skeletonization may cause unnecessary damage to the vascular sheath, small nerve branches, and surrounding tissue. In the present study, all intra-mesenteric vessels were ligated within the fused fascial space regardless of their name. Therefore, the mesocolon and vascular sheath were fully preserved, thus reducing the risk of postoperative diarrhea, celiac disease, SMV injury, as well as lymphatic leakage.

Laparoscopic CME surgery based on developmental anatomy could clearly define the exact boundaries of en bloc resection, leading to a complete mesocolon. However, some limitations should be acknowledged in this study, there was a relatively small sample size, and the detection of MMs and ITCs in the greater omentum by the HE staining and immunohistochemistry analysis might need improvement. Second, this was a retrospective study, which may have resulted in a potential selection bias. Finally, there needed to be more long-term follow-up for oncological outcomes between the two groups in the present study, and we also recollected the short-term follow-up of overall survival results for both two groups of patients. Eventually, thirty-one patients were lost to follow-up due to loss of contact, including 17 patients in the DACME group and 14 patients in the CME group, and the overall survival results in the two groups were not significantly different (eFigure 1 in the Supplement). Therefore, further prospective clinical studies with large samples and long-term follow-up with oncological outcomes are required.

## Conclusion

The greater omentum should be preserved during right-sided colon cancer surgery for patients without serosal invasion. Laparoscopic CME surgery based on developmental anatomy is technically safe and feasible for right-sided colon cancer.

## Electronic supplementary material

Below is the link to the electronic supplementary material.

Supplementary Material 1

Supplementary Material 2

Supplementary Material 3

Supplementary Material 4

Supplementary Material 5

## Data Availability

The datasets generated for this study are available on request to the corresponding authors.

## Abbreviations

CME: Complete mesocolic excision
DACME: Developmental anatomy in laparoscopic complete mesocolic excision
ASA: American Society of Anesthesiologists
BMI: Body mass index
SMV: Superior mesenteric vein
MMs: Micrometastasis
ITCs: Isolated tumor cells
GTH: Gastrocolic trunk of Henle
CVL: Central vascular ligation
