# Supplementary material for: The concept of developmental anatomy: the greater omentum should be resected in right-sided colon cancer?
Source: BMC Surg. 2023 May 17;23:137. doi: 10.1186/s12893-023-02020-8 (PMC10193780; doi:10.1186/s12893-023-02020-8)
Supplement: Supplementary file 5 — Supplementary Material 5 [file 12893_2023_2020_MOESM5_ESM.docx]

**Supplementary Figure**
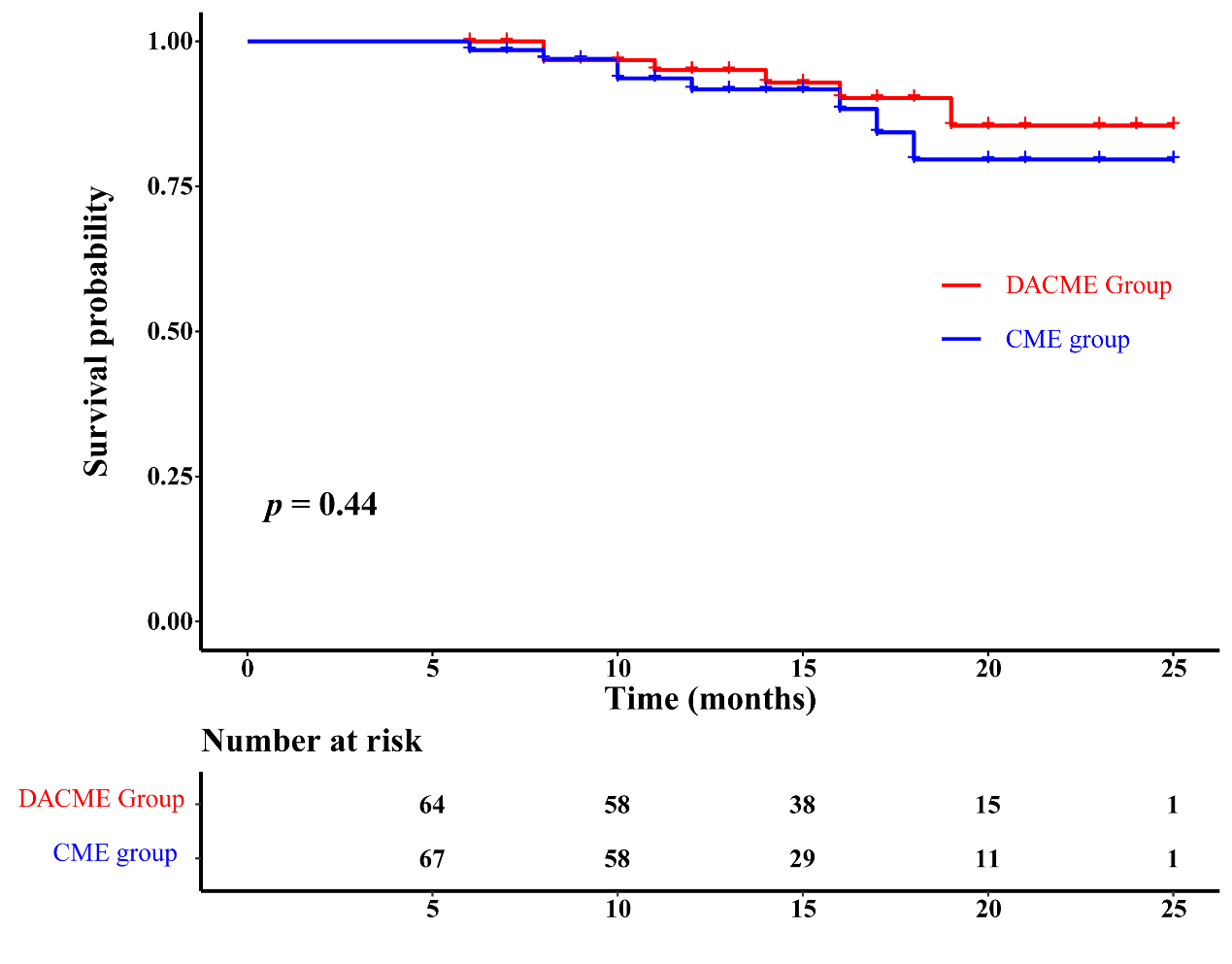
eFigure [1](https://link.springer.com/article/10.1007/s00464-021-08738-x#Fig2): Kaplan–Meier survival analysis for DACME and CME group
